# Multiparametric Intraoperative Ultrasound in Oncological Neurosurgery: A Pictorial Essay

**DOI:** 10.3389/fnins.2022.881661

**Published:** 2022-04-19

**Authors:** Francesco Prada, Riccardo Ciocca, Nicoletta Corradino, Matteo Gionso, Luca Raspagliesi, Ignazio Gaspare Vetrano, Fabio Doniselli, Massimiliano Del Bene, Francesco DiMeco

**Affiliations:** ^1^Department of Neurosurgery, Fondazione IRCCS Istituto Neurologico Carlo Besta, Milan, Italy; ^2^Department of Neurological Surgery, University of Virginia, Charlottesville, VA, United States; ^3^Focused Ultrasound Foundation, Charlottesville, VA, United States; ^4^Acoustic Neuroimaging and Therapy Laboratory, Fondazione IRCCS Istituto Neurologico Carlo Besta, Milan, Italy; ^5^Faculty of Medicine and Surgery, Università degli Studi di Milano, Milan, Italy; ^6^Faculty of Medicine and Surgery, Humanitas University, Pieve Emanuele, Italy; ^7^Department of Neurosurgery, Humanitas Clinical and Research Center, Milan, Italy; ^8^Neuroradiology Unit, Fondazione IRCCS Istituto Neurologico Carlo Besta, Milan, Italy; ^9^Department of Biomedical Sciences for Health, Università degli Studi di Milano, Milan, Italy; ^10^Department of Experimental Oncology, IEO, European Institute of Oncology IRCCS, Milan, Italy; ^11^Department of Pathophysiology and Transplantation, University of Milan, Milan, Italy; ^12^Johns Hopkins Medical School, Baltimore, MD, United States

**Keywords:** intraoperative ultrasound, contrast-enhanced ultrasound, Doppler, B-mode, elastography, glioma, meningioma, metastasis

## Abstract

Intraoperative ultrasound (ioUS) is increasingly used in current neurosurgical practice. This is mainly explained by its affordability, handiness, multimodal real-time nature, and overall by its image spatial and temporal resolution. Identification of lesion and potential residue, analysis of the vascularization pattern, and characterization of the nature of the mass are only some of the advantages that ioUS offers to guide safe and efficient tumor resection. Technological advances in ioUS allow to achieve both structural and functional imaging. B-mode provides high-resolution visualization of the lesion and of its boundaries and relationships. Pioneering modes, such as contrast-enhanced ultrasound (CEUS), ultrasensitive Doppler, and elastosonography, are tools with great potential in characterizing different functional aspects of the lesion in a qualitative and quantitative manner. As already happening for many organs and pathologies, the combined use of different US modalities offers new insights in a multiparametric fashion. In this study, we present the potential of our multiparametric approach for ioUS during neuro-oncological surgery. In this effort, we provide a pictorial essay focusing on the most frequent pathologies: low- and high-grade gliomas, meningiomas, and brain metastases.

## Introduction

### Forward

Image-guided surgery (IGS) is one of the cornerstones of modern neurosurgery. In this context, intraoperative ultrasound (ioUS) is becoming progressively more diffused. Once the shield of bone is removed, the mechanical viscoelastic properties of the brain allow an excellent propagation of US waves and the application of most ultrasonographical techniques. While the main downside is represented by the steep learning curve, ioUS provides a versatile, real-time, close-hand, and multipurpose tool to explore and characterize the operative field beyond a simply morphological perspective (Rubin and Dohrmann, 1983; Newman and Rozycki, 1998; Chacko et al., 2003; Ng and Swanevelder, 2011). A growing body of literature supports IoUS application to achieve gross total removal (GTR) in low-grade and high-grade gliomas, as this parameter is associated with progression-free survival and overall survival improvement (D’Amico et al., 2017). Clinical trials are emerging to evaluate the impact of ioUS on the extent of resection: the first randomized controlled trial on ioUS B-mode was published by Incekara et al. (2021). Furthermore, in recent years, technological advances and pioneering research have been widening the ioUS range of use beyond the mere assessment of the extent of resection.

### Multiparametric Intraoperative Ultrasound

At present, different advanced US techniques are available to provide both anatomical and functional information in real-time during surgery. The complementary application of different US techniques is defined as the multiparametric ultrasound (mpUS) approach. The mpUS approach is an already established technique for the pre-operative and intraoperative evaluation of focal lesions in various organs, especially the liver. For instance, the mpUS in hepatology is used for non-invasive staging of liver fibrosis, detection and classification of portal hypertension and esophageal varices, prognosis in chronic liver diseases, and characterization of focal liver lesions (FLLs) (Grgurevic et al., 2019). Brain ultrasound is going in the same direction (Prada et al., 2016a; Wang et al., 2019; Newsome and Dayton, 2020). In neurosurgery, intraoperative mpUS encompasses gray-scale B-mode, conventional Doppler (color, power, spectral) with ultrasensitive Doppler (usDoppler), strain elastosonography (SE), shear wave elastosonography (SWE), and contrast-enhanced ultrasound (CEUS) with microbubbles (MBs) (Moiyadi, 2016; Pang et al., 2017; Kim et al., 2018; Mannaerts et al., 2018; Grgurevic et al., 2019).

The intrinsic added value of multiparametric imaging is the possibility to exploit each modality to emphasize different characteristics of a lesion, such as its perfusion pattern, its consistency, edema, fibrosis, extension, and relationships, with surrounding structures. In a similar fashion as multiparametric MRI, mpUS aims to give a 360-degree analysis of a lesion with the advantage of being an intraoperative technique with real-time navigation.

B-mode, the oldest and most diffused US modality, is mainly structural imaging. It has achieved an excellent spatial and temporal resolution, even comparable to MRI (Ng and Swanevelder, 2011; Chan et al., 2021), allowing to visualize the tumor, to study the surrounding structures, to assess the extent of resection, and to characterize some aspects of the tumor (e.g., necrosis and solid components) (Prada et al., 2016b; Incekara et al., 2021).

**FIGURE 1:**
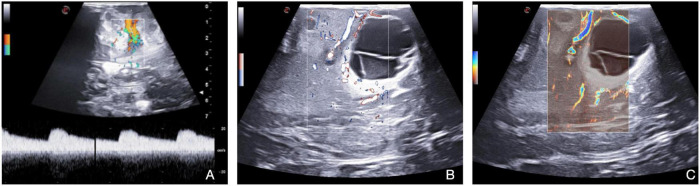
Doppler techniques (Color-Doppler, X-Flow, Micro-V). Color Doppler and spectral Doppler **(A)** bring information about flow direction and velocity with quantification of the velocity through spectral Doppler. X-Flow **(B)** maximizes spatial resolution and Doppler signal penetration capabilities. Micro-V **(C)** permits real-time micro-vascular perfusion analysis, identifying low flow signals.

Doppler imaging (color-, power, spectral-) is the other workhorse of ioUS in neurosurgery. It allows to visualize and characterize the blood flow and perfusion pattern from the arterial side, through the capillary district to the venous collectors of virtually every kind of lesion (Prada et al., 2016b). In this setting, the newest algorithms and instruments permit advanced usDoppler techniques, which enable the operator to visualize and characterize tumoral micro-vascularization. Xflow is a high-resolution, high-definition Doppler technology, which maximizes spatial resolution and Doppler signal penetration capabilities. MicroV represents the state-of-the-art sensitivity for superficial and deeper vessels for real-time microvascular perfusion analysis even in the case of very low flow signal (Figure 1; Yoo et al., 2020; Kloth et al., 2021).

**FIGURE 2:**
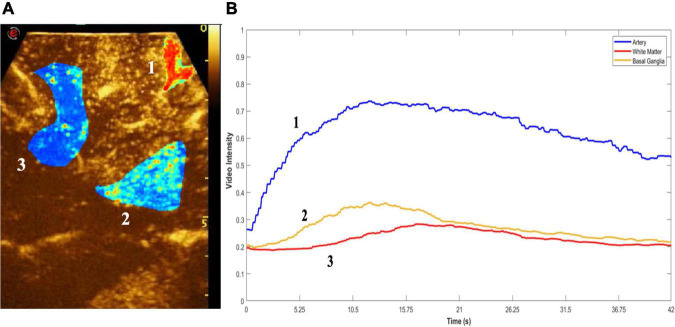
Quantitative CEUS. Quantitative analysis of MBs distribution. **(A)** Region of interest (ROI) is drawn on key structures (1, artery-blue; 2, white matter-red; 3, basal ganglia-yellow), following which peak enhancement and time-intensity curves (TICs) were quantified **(B)**. This enables discernment between different types of brain tumors as well as regions and structures within the brain.

**FIGURE 3:**
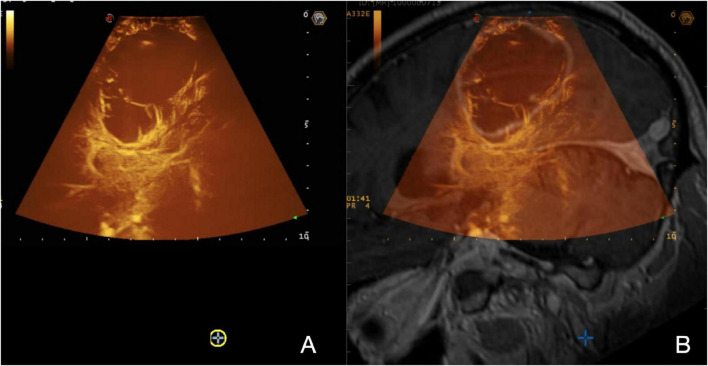
Navigated MIP-CEUS. **(A)** On the left, a real-time MIP-CEUS sequence is displayed; **(B)** on the right, the corresponding pre-operative MRI is fused with real-time ioUS. The signal obtained throughout the MBs distribution can be depicted in a MIP image to describe the entire vascular-tree including smaller vessels under structural and functional aspects, giving detailed representation of the CE pattern.

Among the most advanced techniques, the use of ultrasound contrast agents (UCAs) has opened the door to numerous applications. UCAs dynamics inform on the arterial or venous nature of a vessel, also allowing to characterize the perfusion pattern finally leading to tumor visualization, assessment of the extent of resection, and tumor characterization (Prada et al., 2014a,b, 2016a; Sidhu et al., 2018; Wang et al., 2019; della Pepa et al., 2019; Newsome and Dayton, 2020; Incekara et al., 2021). Notably, the possibility to quantify MBs distribution in the human brain to quantitatively discriminate between tumoral and normal brain tissues has recently been demonstrated (Figure 2; Prada et al., 2021). The application of the CEUS sequence MIP (maximum-intensity projection) permits a detailed evaluation of intratumoral vascularization (Figure 3). In some preclinical studies, CEUS seems to even permit a real high-definition “acoustic angiography” to visualize the microvascular neo-angiogenesis of tumors (Newsome and Dayton, 2020). The use of CEUS during neuro-oncological procedures has been included in the guidelines from the European Federation of the Societies for Ultrasound in Medicine and Biology (EFSUMB) in 2017, representing a paradigm shift for the use of US in neurosurgery (Sidhu et al., 2018).

**FIGURE 4:**
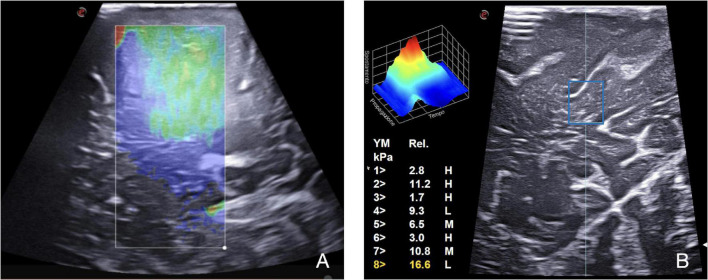
SE and SWE. Elastosonography provides information regarding the tumor and surrounding parenchyma mechanical properties through qualitative and quantitative evaluation. **(A)** On the left, SE in a case of metastasis: a mechanical force is applied to measure lesion stiffness, which is expressed on a color scale for qualitative assessment. **(B)** On the right, SWE, using the QElaxto algorithm, provides a quantitative assessment of stiffness of a specific ROI inside the lesion.

Another fundamental technique for an exhaustive evaluation of a lesion is elastosonography. It can provide details about the mechanical properties of the tumor compared with the parenchyma of the surrounding normal brain. For instance, the use of algorithms based on elastosonography appears to be more accurate than those from B-mode in differentiating intraoperatively HGGs and metastasis (Cepeda et al., 2021b). Technically, two methodologies, namely, strain elastography (SE) and shear wave elastography (SWE), are available. SE applies a mechanical force to measure lesions’ stiffness, which is expressed on a color scale for qualitative assessment. Differently, SWE provides a quantitative assessment. This technique exploits a US stimulus to induce tissue displacement, thus providing a quantitative representation of the stiffness. In the current practice, elastosonography seems useful mainly in gliomas and meningiomas characterization (Figure 4; Prada et al., 2015, 2016b,^2019^; della Pepa et al., 2020; Cepeda et al., 2021a; Chan et al., 2021; Menna et al., 2021; Yin et al., 2021).

Each ioUS technique has not only advantages but also limitations (Šteňo et al., 2021), which foster the need for a multiparametric approach. IoUS has still a low level of evidence at this time (Fountain et al., 2021), although it has an interesting potential not only in characterizing the lesion but also in permitting quantitative measurements, which are important tools for research, laying the foundations for algorithm-based characterization of brain lesions (Kim et al., 2018; Wang et al., 2019; Prada et al., 2021).

## Ultrasound Equipment and Acquisition Modalities

Intraoperative ultrasound in neuro-oncological surgery is dynamic and requires tailoring of the US settings and probes several times during the operation. For instance, various probes exist to address almost every situation: high-frequency probes for superficial tumors, linear or convex multifrequency probes for more deep-seated lesions, micro-convex or hockey-stick probes for exploring surgical cavities, and dedicated pituitary probes (Prada et al., 2016b; Cabrilo et al., 2021). The spectrum of available US techniques depends both on the probe employed and on the scanner to which they are connected. In everyday practice, we mainly use the last generation US device (MyLab 9, Esaote, Genoa, GE, Italy) with an integrated magnetic fusion imaging system for virtual navigation (MedCom GmbH, Darmstadt, DE, Germany). Concerning the probes, our workhorses are a 3–11 Hz linear probe and a micro-convex intracavitary probe (Prada et al., 2016b).

After the craniotomy and before the dural opening, the probe is wrapped in a sterile plastic sheath with coupling sterile US gel in standard fashion. The first US acquisition is usually performed *trans-*durally for a preliminary inspection of the operative field, landmarks identification, lesion localization, and characterization and finally to identify the best surgical corridor to the lesion. The surgical field is continuously irrigated with sterile saline solution to allow US coupling and to reduce artifacts related to air or blood clots. Multiple US scans are performed throughout the surgery not only to guide surgical removal but also to assess the extent of resection, perfusion changes, or complications occurrence. A standard multiparametric US exam is based on the following modalities. B-Mode is usually the first employed technique, as it allows a high-resolution structural characterization of lesions and surrounding structures. Conventional Doppler techniques are used for main vessels identification: color- and spectral-Doppler are combined to identify and characterize whether the blood flow is present. Ultrasensitive usDoppler techniques (Xflow, MicroV, Esaote, Genoa, GE, Italy) are employed to study the perfusion pattern and capillary angio-architecture of the tumor and surrounding parenchyma. Elastosonography provides information regarding the tumor and surrounding parenchyma mechanical features through qualitative and quantitative evaluation (SE, SWE). Finally, CEUS, relying on UCAs (Sonovue), allows to observe tumor and parenchyma perfusion patterns, characteristically recognizing 4 phases: “wash in” representing the arterial signal, “parenchymal perfusion” giving an overall idea about capillary bed perfusion distribution, “peak” which is the maximum contrast intensity associated with tumor perfusion, and “wash out” corresponding to venous outflow. The signal obtained throughout the four phases can be depicted in a MIP image to describe the entire vascular tree including smaller vessels under structural and functional aspects.

At this point, neurosurgeons proceed in lesion removing, practicing multiple multiparametric scans for a real-time evaluation of the neighboring structures, residual tumor, and changes occurrence.

Once the lesion is completely removed, a final multiparametric scan is performed to evaluate potential hidden residual tumor and potential complications occurrence.

In the following paragraph, we sought to summarize the main applications, values, and issues, together with our considerations concerning the use of multiparametric ioUS in the four, most relevant, entities in oncological surgery, namely, low-grade gliomas (LGGs), high-grade gliomas (HGGs), meningiomas, and brain metastases.

## Low-Grade Glioma

**FIGURE 5:**
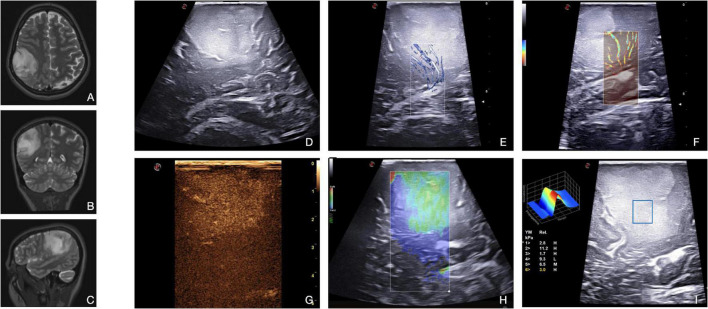
Illustrative case of a diffuse astrocytoma (Grade 2 WHO, 2021). **(A–C)** Pre-operative MRI demonstrates a right frontal-temporal-parietal low-grade, non-enhancing intra-axial tumor. **(D)** In B-mode, LGG appears as a hyperechoic diffuse mass with blurred margins. Both X-flow Doppler **(E)** and Micro-V **(F)** demonstrate the venous drainage toward the ependyma. CEUS **(G)** allows to differentiate peritumoral edema from tumoral tissue. Typically, LGG shows a mild, dotted CE with homogenous pattern. SE **(H)** and SWE **(I)** allow a qualitative and quantitative evaluation, respectively, of the stiffness of the lesion. This information can be exploited to identify the interface between tumor and brain as shown in image **(H)** (the lesion appears green-softer than the parenchyma beneath).

Low-grade glioma (LGG, WHO, 2021 grade 2) (Louis et al., 2021) represents a real challenge for neurosurgeons. LGGs are hardly discernible from surrounding healthy brain parenchyma at macroscopic inspection. Likewise, as these lesions do not show contrast enhancement at MR imaging, the definition of surgical margins is most often left to FLAIR-hyperintense areas representative of neoplastic cells’ infiltration and perilesional vasogenic edema. In our experience, in B-Mode, LGGs appear as slightly hyperechoic owing to homogeneous echo signals with blurred margins (Figure 5). Therefore, the distinction of neoplastic infiltration from perilesional edema remains an open challenge (Solheim et al., 2010; Prada et al., 2014a,b; del Bene et al., 2018). However, several papers have demonstrated the positive impact of B-mode US on the extent of resection in LGGs (Sastry et al., 2017; Zhang et al., 2017; del Bene et al., 2018; Munkvold et al., 2018; Mazzucchi et al., 2020). The issue of artifacts occurrence in intra-axial brain tumor surgery should be mentioned. This phenomenon can lead to the impossibility of distinguishing between tumor residue and artifact related to surgical manipulation (Šteňo et al., 2021).

Contrast-enhanced US highlights the perfusion pattern of the tissues, allowing to identify not only neoplastic areas, where vessels are more represented, but also higher grade spots, such as anaplastic foci, thus guiding surgical decision-making (Prada et al., 2014a). In particular, the parenchymal phase of CEUS enhancement can be helpful in the identification of residual tumors depending on the specific perfusion pattern of the tumor (Prada et al., 2016a). As presented in Figure 5, LGGs show a mild, dotted contrast-enhanced image with a diffuse appearance. The pattern is homogenous as there are no necrotic cores or cystic and hemorrhagic areas, while arterial, parenchymal, and venous phases are slower than high-grade gliomas. These considerations may allow an intraoperative glioma WHO grade characterization even in dubious cases (Prada et al., 2014b,^2021^; Cheng et al., 2016; del Bene et al., 2018; Wu et al., 2019).

In LGGs surgery, conventional Doppler techniques are exploited to visualize main neighboring vessels in strict relationship with the tumor. With color- and spectral-Doppler, it is possible to define the flow velocity and to distinguish arteries from veins. Concerning tumor perfusion patterns, it is not possible to identify tumoral macrovessels in LGGs. Xflow and MicroV Doppler can explore the micro-vasculature, which in LGGs is usually scarce (Figure 5). For this reason, in the case of LGGs, usually CEUS is much more informative than Doppler techniques (Ng and Swanevelder, 2011; Prada et al., 2014b,^2015^; Cepeda et al., 2021b).

Finally, elastosonography is another useful US tool to characterize the nature and to visualize the lesion. LGGs, in most cases, appear stiffer than the surrounding healthy brain as shown in Figure 5. This characteristic can help the neurosurgeon in defining its borders and eventually post-resection residue (Chauvet et al., 2016; Prada et al., 2019; Chan et al., 2021; Yin et al., 2021).

In the case of oligodendrogliomas, specific considerations must be made: B-mode scans are often characterized by intratumoral calcifications depicted as highly echogenic spots. CEUS shows faster contrast enhancement (CE) and better-defined borders than other LGGs, and all the phases are slower than that in anaplastic astrocytomas. CE patterns are homogeneous even with the presence of intralesional calcification and cysts (Prada et al., 2014b; del Bene et al., 2018).

Multiparametric approach provides the surgeon with last-generation instruments to intraoperatively visualize, characterize, and identify lesions and surrounding structures. In our practice, combining different techniques is preferred to standard B-mode alone, as this allows to predict tumor biology and to better visualize tumor, relationships, and residues, which could lead to safer and more effective surgery: despite the potential of mpUS, more trials are needed to demonstrate cost-effectiveness benefits from this approach (Prada et al., 2014a, 2016b; Cheng et al., 2016; del Bene et al., 2018).

## High-Grade Glioma

**FIGURE 6:**
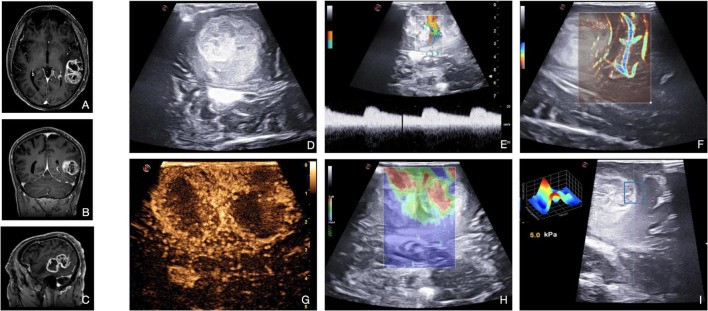
Illustrative case of glioblastoma (Grade 4 WHO, 2021). **(A–C)** Pre-operative MRI demonstrates a left temporal high-grade, enhancing intra-axial tumor. **(D)** B-mode provides mainly structural information on the lesion and on the main relationships with surrounding structures. B-mode shows the presence of a vast hypoechoic necrotic core within the lesion. Color-Doppler **(E)** and MicroV **(F)** depict the main vessels and create a map of the vascular tree. Spectral-Doppler characterizes the flow inside a vessel, thus providing information on the nature and relevance: in panel **(E)** an artery between the two lesion’s necrotic core with a flow velocity of 20 cm/s. MicroV highlights the surrounding vessels and the smaller capillaries inside the lesion. CEUS **(G)** provides a representation of the anatomical and functional architecture of the entire vascular tree. The image acquired at the peak phase is suggestive for the entity of perfusion of HGG, also dissecting the components of the lesion: two intense CE ring-shaped areas of vital tissue delineating two central low CE necrotic cores. Qualitative **(H)** and quantitative **(I)** elastosonography inform on the stiffness of the different components of the lesion (e.g., necrosis vs. vital tissue) and on the interface between tumor and brain. SE shows two soft areas (red) surrounded by stiffer areas (green-blue). SWE, using the QElaxto algorithm, can define the stiffness of a specific ROI inside or outside the lesion.

High-grade gliomas (WHO, 2021, 3 and 4) (Louis et al., 2021) are easily recognizable in intraoperative B-mode as their appearance is inhomogeneous due to their necro-cystic or hemorrhagic component, their irregular shape, and unclear borders. In our experience, HGGs are usually characterized by hyperechoic margins mixed with iso-ipoechoic central necrotic areas and infiltrative patterns (Figure 6). In general, the margins are more identifiable than that in LGGs even it is still difficult to differentiate between tumor boundaries and peri-lesional edema. However, B-mode is highly informative in HGG resection as demonstrated in numerous papers (Chacko et al., 2003; Solheim et al., 2010; del Bene et al., 2018; Carai et al., 2021; Incekara et al., 2021; Yin et al., 2021). At first, it allows a real-time anatomical study of the lesion, surrounding structures, and tumor architecture even before the dural opening. Furthermore, as the dural opening is accompanied by a brain shift, the anatomy could be modified. B-mode is used to navigate again to identify the lesion, the anatomical landmarks, and the vital structures to be preserved. In addition, the neurosurgeon can observe which gyrus is infiltrated and thus tailoring the corticectomy preserving the spared gyrus. During surgery, B-mode US can be repeated multiple times to guide the surgery and estimate the extent of resection (Solheim et al., 2010; Prada et al., 2014b, 2016b; del Bene et al., 2018; Incekara et al., 2021; Yin et al., 2021).

Doppler ultrasound is applied to acquire more information about the vascularization of the lesion and the relationships with the surrounding vessels. In addition, it can be helpful for the orientation of the surgical field and the preparation of the corridor. While spectral-Doppler and color-Doppler are used to identify the vessels and understand their arterial or venous nature, power-Doppler is exploited to better highlight the smaller branches from the surrounding vessels (Figure 6). However, conventional Doppler alone gives limited information (Prada et al., 2016b,^2018^; Šteňo et al., 2016). Application of usDoppler (Xflow, MicroV) is particularly useful for the surgeon, as the sensitivity and high-definition of these techniques permit to visualize and estimate more accurately HGG’s smallest vessels and the entity of neoangiogenesis (Figure 6).

Elastosonography demonstrates HGGs to be usually softer than the healthy brain parenchyma (Figure 6), thus helping to discern between HGGs and LGGs (Cepeda et al., 2021b). Quantitative analysis made possible by SWE is particularly interesting for the creation of algorithms to discern intraoperatively the nature of the lesion, such as distinguishing HGGs from solitary brain metastases (Prada et al., 2016b,^2019^; Cepeda et al., 2021b; Yin et al., 2021). SE and SWE are useful even for assisting tumoral resection (Newman and Rozycki, 1998).

Finally, CEUS allows a real-time angiosonography of the HGG which provides an in-depth analysis of the anatomical and functional organization of the vascular tree, also identifying their relationship with the neighboring arteries and veins. As shown in Figure 6, HGGs commonly display intense CE signal characterized by fast perfusion patterns. The arterial phase shows macro-vessels supplying the mass and a peripheral enhancement that moves toward the inner areas of the lesion. While the proliferating areas have high CE, the necrotic areas don’t show any. The MIP sequence emphasizes the smaller vessels, giving a detailed representation of this CE pattern. As regards glioblastomas (GBM, WHO, 2021 grade 4), there are generally two different CE patterns: the peripheral CE surrounding a necrotic core or a more nodular and heterogeneous high CE within the lesion alternated by small spots of necrosis (Ng and Swanevelder, 2011; Prada et al., 2016a; Sidhu et al., 2018; Cepeda et al., 2021a; Menna et al., 2021).

In general, HGG borders are always better visualized after MBs administration than in B-mode due to the different vascularization of the tumor with respect to the normal brain tissue (Schrope and Newhouse, 1993). Thanks to these characteristics, CEUS has been exploited to identify tumor remnants after resection in different studies: CEUS shows persistent CE in nodular tissue around the surgical cavity, while B-mode alone can be unclear (Ng and Swanevelder, 2011; Fountain et al., 2021; Menna et al., 2021).

## Meningioma

Most intracranial meningiomas are benign tumors with a good prognosis after standard treatment. However, under structural and functional aspects they may differ enormously, also contracting threatening relationships with vital structures (Solheim et al., 2009). MpUS provides valuable information for tumor identification, enabling analysis of the tumor location, relationships, mechanical properties, and vascularization (Prada et al., 2015; Menna et al., 2021).

**FIGURE 7:**
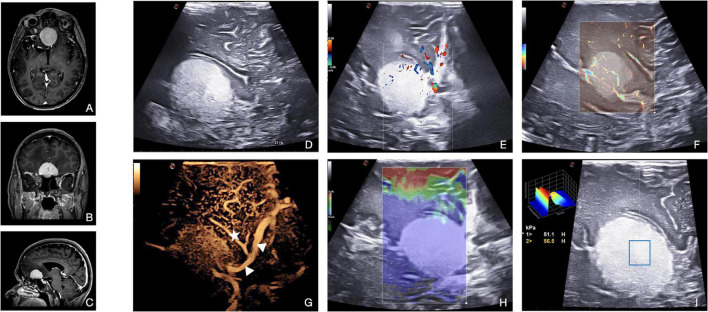
Illustrative case of atypical meningioma (Grade 2 WHO, 2021). **(A–C)** Pre-operative MRI demonstrates a Planum Sphenoidale, enhancing, extra-axial tumor. **(D)** Standard B-mode: meningioma appears hyperechoic, compared with normal brain parenchyma, with a homogeneous pattern and a granular aspect. Tumor margins are sharply defined. **(E)** Color- and spectral-Doppler show the presence, direction, and velocity of flow of the vessels of the anterior circulation. **(F)** Micro-V depicts also slow flow in small-diameter vessels within the lesion, providing information about the entity of perfusion/vascularization. **(G)** CEUS enhances the findings of Doppler modalities representing a peritumoral and intratumoral vascular tree. In this patient, we can appreciate the relationships between meningioma and anterior cerebral artery (arrowheads) and Heubner’s artery (star). The basal portion of the lesion cannot be properly studied due to calcification within the meningioma itself. In meningioma surgery, elastosonography informs on lesion mechanical features, thus guiding surgical decision-making. In this case, **(H)** SE shows meningioma with a higher stiffness than the surrounding tissues. **(I)** SWE confirms the higher stiffness also providing a quantitative evaluation: Young’s modules in the ROI of 56 kPa.

In our experience, meningiomas are generally recognizable in B-mode as hyperechoic lesions with a homogeneous pattern, a granular aspect, and fine trabeculation (WHO 1-2) (Figure 7) or with several hypoechoic areas of necrotic degeneration (WHO 3). When present, intratumoral calcifications are depicted as highly echogenic components. In addition, meningioma margins are generally well defined even though, especially in the case of brain invasion, surrounding brain parenchyma can also be hyperechoic (Prada et al., 2015, 2016b). Through the careful interpretation of the B-mode signal, it is possible to achieve an initial overview of tumor mechanical features, of the nature of the interfaces and relationships, and of gross extension. Traditional color- and spectral-Doppler show the presence, direction, and velocity of the flow in surrounding or encased large vessels, enabling the surgeon to rapidly create a real-time flow map to avoid them during surgery. This consideration is particularly true in the case of deep-seated meningiomas close to basal veins, vital arteries, or in meningiomas invading or strictly adherent to dural sinuses. Solheim et al. (2009) examined the application of power-Doppler in meningioma surgery concluding that in most cases, this US modality could be helpful in visualizing feeding arteries and adjacent important vessels leading to safe preservation of them. Anyway, the authors recognized this technique to be limited by the difficulty to study low-flow vessels. Conversely, micro-Doppler (Xflow, MicroV) magnifies slow flow also in smaller vessels, providing the surgeon with information about the entity of meningioma vascularization and location of main feeders, which can be useful to plan resection (Figure 7).

Contrast-enhanced ultrasound allows a reliable intraoperative visualization of the peritumoral and intratumoral vascular tree, providing real-time dynamic images of intrinsic tumor perfusion, visualizing “wash in” velocity, parenchymal perfusion, and peak and venous “wash out.” Thanks to the most recent algorithms for quantitative CEUS, we expect its intraoperative application to change meningioma surgery in the near future (Prada et al., 2021). Usually, the CEUS pattern for meningiomas consists of an intense and rapid contrast enhancement with an impressive peak, especially for higher grades, a slow venous, and not always visible (Prada et al., 2015). MIP on the CEUS image sequence can be used to obtain better visualization of intra-tumoral vascular tree and higher definition of the lesions’ borders, even allowing a better understanding of the relationship between tumors and surrounding vessels in relationship with it (Figure 7). CEUS in meningioma surgery offers two main advantages. The boundaries of the lesion are easily identified, also enabling easier detection of residual tissue. Second, the main arterial feeders and surrounding vessels are visualized and characterized, thus providing pivotal information for tumor debulking and resection. These data can be valuable in guiding surgical decision-making and planning, for instance, by targeting the arterial feeders first to obtain a complete tumor devascularization before debulking. In addition, CEUS might be repeated to evaluate the degree of remaining vascularization from other feeders outside the main dural supply.

Meningiomas encompass quite a wide spectrum of textures and brain-tumor interfaces which unavoidably play a fundamental role during resection; however, MR imaging is not always predictive of these aspects. Elastosonography instead, by directly calculating the lesions’ Young modulus, provides real-time, intuitive imaging of meningiomas’ stiffness and relation to surrounding structures. From our experience, this feature plays a pivotal role in the planning of resection strategy, to the point of influencing the choice of a particular instrument or approach (Figure 7; Prada et al., 2015; della Pepa et al., 2020).

## Brain Metastases

**FIGURE 8:**
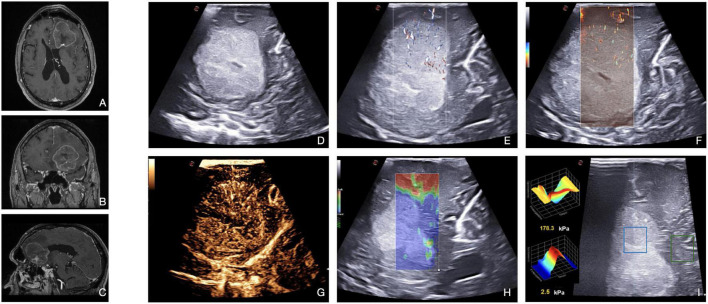
Illustrative case of brain metastasis of lung carcinoma. **(A–C)** Pre-operative MRI demonstrates a left frontal, enhancing, intra-axial tumor. **(D)** B-mode: the lesion appears distinctly hyperechoic with a diffuse granular and heterogeneous aspect. The central core is necrotic. Tumor/brain interface is recognizable. **(E,F)** Micro-Doppler techniques (X-Flow and micro-V) demonstrate the presence of intratumoral microvascularization, thus informing on perfusion pattern. **(G)** CEUS shows a moderate and heterogeneous contrast enhancement pattern with centripetal dynamics. The central necrotic core is highlighted. In general, metastasis may appear as stiffer or milder than a healthy brain. In this case, **(H,I)** SE shows a high stiffness (blue) in the neoplastic region analyzed, while SWE quantifies the stiffness in specific ROI (the central blue ROI has a stiffness of 2.5 kPa, while the peripheral green ROI of 178.3 kPa).

Brain metastases are generally recognizable in B-mode as distinctly hyperechoic lesions with a diffuse granular or heterogeneous aspect (e.g., peripheral ring and central necrosis or nodule and cystic areas). The most frequent B-mode pattern encompasses solid components and cystic or necrotic areas varying in size and owing well-demarcated boundaries even in the case of surrounding edema (Figure 8).

Conventional Doppler techniques allow to visualize main neighboring vessels and to characterize the blood flow features. Xflow and MicroV techniques usually depict relevant perfusion at the capillary level through several centripetal feeders from the surrounding parenchyma.

Then, CEUS defines a rapid contrast enhancement, with a fast arterial phase and contrast enhancement peak. Contrast enhancement is intense and persistent with a delayed venous phase and irregular and heterogeneous CE pattern (Figure 8). CEUS depicts with detail the nature of the tissues (e.g., necrotic vs. vital) in virtue of the degree of CE. The arterial supply is frequently centripetal with many macrovessels within the lesion, whose visualization can be magnified through MIP sequence. As such, CEUS can aid brain metastases resection by identifying feeding arteries, draining veins, and tumor vasculature.

Elastosonography allows to characterize the mechanical properties of the lesion and to plan the surgical approach. In general, brain metastases may be stiffer (kidney, colon) or softer (lung, endometrial) than a normal brain. As described by Chauvet et al. (2016), brain metastases have a low mean elasticity value–partially due to central necrosis. Furthermore, elastosonography can guide the differential diagnosis between metastasis and HGGs (Prada et al., 2016a; Wu et al., 2019).

In particular, in bran metastases, IoUS plays a major role at the beginning of the resection, to understand the relationships and the feeders and to orientate in the surgical field, or in case of piecemeal resections when en-bloc removal is not feasible, to assess the extent of resection and residue presence.

## Multiparametric Ultrasound Limitations

Although mpUS owns great potential, certainly it is limited by some relevant aspects which should be taken into account. The learning curve of mpUS is certainly steep or even steeper if compared with standard B-mode. Specific courses and resources should be promoted and supported. Research in this field is still at the dawn, thus still not allowing to demonstrate the real reach of this approach. Another intrinsic limitation of this approach is the necessity of the last generation US device, which hinder the spreading of the technique. Each modality employed in multiparametric evaluation has specific limitations that should always be considered when evaluating the reliability of this methodology. As for standard applications of US, acoustic enhancement artifacts (AEAs) are a major issue that could hamper the visualization of tumor borders and residue presence after debulking. To overcome this problem, new approaches are under investigation, such as the use of dedicated coupling fluids, micro-probes to study the cavity from inside, CEUS applications, navigated US, and the exploiting of surrounding healthy brain to study the surgical cavity. Finally, being neurosurgery a new field of application of mpUS, the level of evidence for this approach is still low, and further investigations are warranted to exploit the full potential (Solheim et al., 2010; Prada et al., 2016b; Šteňo et al., 2016, 2021; Unsgård et al., 2019).

## Conclusion

Ultrasound brain imaging applications have been historically limited by the presence of bone shielding, thus shifting diagnostics and research to CT and MRI applications. As a matter of fact, a craniotomy opens a window that allows US imaging of the brain as for other US accessible body organs where multiparametric US has been exploited for several years to obtain structural and functional information about tumoral biology before histopathological examination. IoUS has many advantages in neuro-oncological surgery. Due to the multiparametric approach, it is possible to study the tumor and the surrounding parenchyma under structural and functional aspects in real-time and dynamically during the operation. The different ioUS techniques can be exploited both in malignant and benign tumors providing the operators with crucial information to optimize surgery, identifying the remnants, and facilitating maximal resection. The impressive spreading of ioUS among different neurosurgical centers all over the world testifies the potential of multiparametric ioUS in neuro-oncological surgery. To this end, we sought that every craniotomy performed, not only for brain tumors but also for other neurosurgical cases, such as trauma, epilepsy, or vascular cases, will be used as an opportunity to better understand brain physiopathology in real-time. Closing up the patients with prosthesis that allow for post-operative imaging, and eventually therapy, will allow to carry this opportunity in a post-operative setting as well (del Bene et al., 2022). Nevertheless, further research and efforts regarding improvements, training, standardization, and reproducibility of intraoperative ultrasound modalities as stand-alone tools and in a multiparametric fashion are still required.

## Author Contributions

FP contributed to acquisition of data, study concept, and design. FP and MD contributed to critical revision of the manuscript for intellectual content. RC and NC contributed to original draft preparation. FP, RC, NC, MG, LR, FD, IV, MD, and FDM contributed to reviewing and editing. All authors contributed to the article and approved the submitted version.

## Conflict of Interest

The authors declare that the research was conducted in the absence of any commercial or financial relationships that could be construed as a potential conflict of interest.

## Publisher’s Note

All claims expressed in this article are solely those of the authors and do not necessarily represent those of their affiliated organizations, or those of the publisher, the editors and the reviewers. Any product that may be evaluated in this article, or claim that may be made by its manufacturer, is not guaranteed or endorsed by the publisher.
